# Context-Specific Metabolic Networks Are Consistent with Experiments

**DOI:** 10.1371/journal.pcbi.1000082

**Published:** 2008-05-16

**Authors:** Scott A. Becker, Bernhard O. Palsson

**Affiliations:** Department of Bioengineering, University of California San Diego, La Jolla, California, United States of America; University of Washington, United States of America

## Abstract

Reconstructions of cellular metabolism are publicly available for a variety of different microorganisms and some mammalian genomes. To date, these reconstructions are “genome-scale” and strive to include all reactions implied by the genome annotation, as well as those with direct experimental evidence. Clearly, many of the reactions in a genome-scale reconstruction will not be active under particular conditions or in a particular cell type. Methods to tailor these comprehensive genome-scale reconstructions into context-specific networks will aid predictive in silico modeling for a particular situation. We present a method called Gene Inactivity Moderated by Metabolism and Expression (GIMME) to achieve this goal. The GIMME algorithm uses quantitative gene expression data and one or more presupposed metabolic objectives to produce the context-specific reconstruction that is most consistent with the available data. Furthermore, the algorithm provides a quantitative inconsistency score indicating how consistent a set of gene expression data is with a particular metabolic objective. We show that this algorithm produces results consistent with biological experiments and intuition for adaptive evolution of bacteria, rational design of metabolic engineering strains, and human skeletal muscle cells. This work represents progress towards producing constraint-based models of metabolism that are specific to the conditions where the expression profiling data is available.

## Author Summary

Systems biology aims to characterize cells and organisms as systems through the careful curation of all components. Large models that account for all known metabolism in microorganisms have been created by our group and by others around the world. Furthermore, models are available for human cells. These models represent all possible biochemical reactions in a cell, but cells choose which subset of reactions to use to suit their immediate purposes. We have developed a method to combine widely available gene expression data with presupposed cellular functions to predict the subset of reactions that a cell uses under particular conditions. We quantify the consistency of subsets of reactions with existing biological knowledge to demonstrate that the method produces biologically realistic subsets of reactions. This method is useful for determining the activity of metabolic reactions in *Escherichia coli* and will be essential for understanding human cellular metabolism.

## Introduction

Commonly, genome-scale metabolic networks are reconstructed to contain all known metabolic genes and reactions in a particular organism [1]. These reconstructions are thus a superset of the metabolic reactions that are functioning in the organism at any one time. The processes that determine which enzymes are active in a cell are often overlooked in constraint-based studies. Of particular interest are the transcriptional regulatory processes in cells which choose a subset of possible enzymes for activity at any given time.

Knowledge of transcriptional regulation of metabolism comes from different sources. At a low level, from the bottom up, some of the regulatory proteins that control the transcription of sets of metabolic genes are known [2]. At a higher level, from the top down, gene expression data provides a picture of what genes are being transcribed at a particular time, and hence which enzymes are probably active in the cell [3]. Both of these types of knowledge can be used to refine metabolic networks under given conditions.

There are three ways to study how regulation tailors gene-expression under a specific condition. First, if a transcriptional regulatory network network (TRN) is available, then the transcription state of the cells can be computed for a given input [4],[5]. However, genome-scale TRNs are not available. Even for *Escherichia coli*, it has been estimated from dual perturbation experiments that only about one-fourth or one-third of its TRN is currently known [6]. ChIP-chip data and other approaches may soon enable more comprehensive reconstructions. Second, in the absence of a TRN, optimization procedures based on the assumption that the organism picks out the best set of reactions to meet a physiological objective have been used [7]. However, there are multiple solutions to such problems [8] and no real way to determine which internal reactions are used in the absence of flux data. In addition, the statement of an objective introduces a ‘user-bias’ and such objective may not actually be relevant to the true physiological state.

The third approach to study regulation relies on the available of expression profiling data. If such data is available for the conditions being examined we can directly examine the expression of the ORFs accounted for in a genome-scale reconstruction. Metabolic network reconstructions can be combined with gene expression data from different states to identify regulatory principles in organisms [9]. Pathway-based analysis methods can be used to predict the usage of entire pathways of reactions based on the expression state of multiple genes [10]. Gene expression data has previously been applied with yeast to predict which reactions may be inactive on a gene-by-gene basis [11]. More recently, gene expression data has been interpreted in terms of elementary modes [12], moving towards a more functional view of analysis.

The results of these methods are dependent on the quality of the expression data that is used as input. Expression data is known to be noisy, and the variety of methods for converting the fluorescence intensity of thousands of spots on a chip to semi-quantitative readings of mRNA molecule counts do not produce equivalent results [13],[14]. Importantly, due to the noise, it is impossible to define a comprehensive set of present mRNA transcripts without a large number of false-positives. Practically speaking, one can say either (1) these few mRNAs are almost certainly present in the cell, or (2) some of these many mRNAs are maybe present in the cell. Pathway-based methods, for example [10], attempt to avoid the noise problem by assuming that all mRNAs assigned to a particular pathway should be present or absent together. This is dependent on biased, human-imposed pathway definitions, and reactions that function within a pathway can also function outside of that pathway, limiting the use of such assumptions.

Here we use gene expression data in combination with objective functions to create functional models despite potentially noisy data. We describe the use of genome-scale transcriptomic data to constrain reactions in both bacteria and human cells, enabling context-specific metabolic networks to be reconstructed and compared. We quantitatively define the consistency of gene expression data with assumed functional states of a cell, demonstrating agreement with physiological data. Context-specific metabolic networks will be virtually essential to accurately model human metabolism due to the variety of cell types and their corresponding metabolic processes.

## Results/Discussion

### GIMME Algorithm

**Figure 1:**
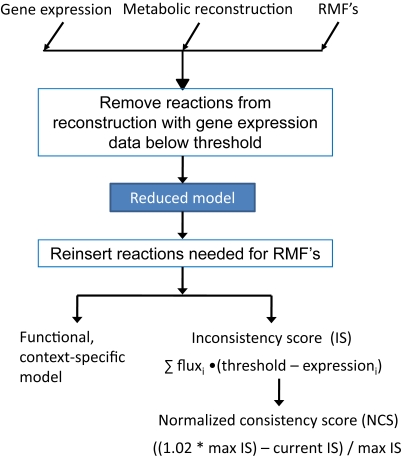
A flow chart schematic representation of the GIMME algorithm. The GIMME algorithm takes three inputs: gene expression (or any other data type) mapped to reactions, a metabolic reconstruction, and one or more RMFs. A metabolic reconstruction is mapped through a data set, removing reactions that are not available and creating a reduced model. Reactions are reinserted into the reduced model as needed to achieve RMFs (such as growth and/or ATP production), resulting in a functional, context-specific model that features minimal disagreement with the data. The consistency score quantifies the disagreement with data, showing the minimal sum of fluxes weighted with reaction data deviations from data.

The approach to the construction of context-specific metabolic networks is termed Gene Inactivity Moderated by Metabolism and Expression (GIMME) and is illustrated in Figure 1. As inputs, the algorithm requires: (1) a set of gene expression data, (2) the genome-scale reconstruction, and (3) one or more Required Metabolic Functionalities (RMF) that the cell is assumed to achieve. Preliminary tests (not shown) suggest that proteomic data can be substituted for expression profiling data. Given these three inputs the algorithm produces a list of reactions in the network that are predicted to be active and an inconsistency score (IS) that quantitatively classifies the disagreement between the gene expression data and the assumed objective function. This inconsistency score is converted to a normalized consistency score (NCS), allowing for relative comparisons of how well each gene expression data set agrees with a particular metabolic function.

**Figure 2:**
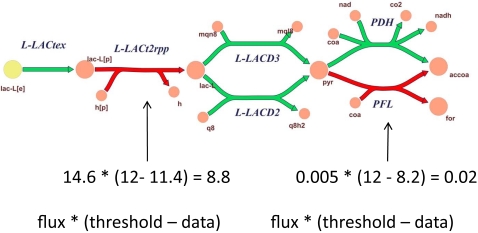
The computation of inconsistency scores. Inconsistency scores for each reaction are computed by multiplying the deviation from a threshold by the required flux through a reaction. In the example here, the green reactions have data above the threshold, set to 12 (this is a parameter; see text). The red reactions have data below the threshold (11.4 and 8.2). The calculation of the inconsistency score corresponding to each reaction is shown numerically as flux multiplied by the deviation from the cutoff. They each increase the inconsistency score, implying that the data are less consistent with the objective of growing on lactate. Greater required fluxes and greater deviation from the threshold both increase the inconsistency scores. The total inconsistency score is the sum of all individual reaction scores.

Simply speaking, reactions that correspond to mRNA transcript levels below a specified threshold are tentatively declared inactive. If the cell cannot achieve the desired functionality without at least one of these reactions, linear optimization is used to find the most consistent set of reactions to reactivate. Inconsistency scores are calculated based on the product of distance from threshold and necessary flux for each reaction required to be reactivated, as illustrated in Figure 2. A smaller inconsistency score indicates that the data is more consistent with the RMF. The GIMME algorithm produces the network with the minimal inconsistency score through the following two-step procedure:

- Find the maximum possible flux through each RMF (allowing usage of all reactions).
- Constrain the RMF's to operate at or above some minimum level (generally a percentage of the maximum found in [A]) and identify the set of available reactions that best fit a quantitative data set.

Part A is achieved through flux balance analysis (FBA) [15]. Part B involves the solution of the following linear programming problem:
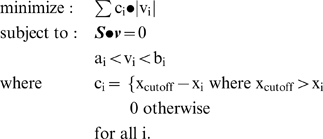
In the formulation, x_i_ is the normalized gene expression data mapped to each reaction. x_cutoff_ is a cutoff value set by the user above which a reaction is definitely present; there is no contribution to the inconsistency score from reactions that are above this threshold. **S** is the stoichiometric matrix with reactions as columns, metabolites as rows, and stoichiometric coefficients as elements. **v** is the flux vector, quantitatively describing the flow through each reaction. a_i_ and b_i_ are the lower and upper bounds, respectively, for each reaction and define the minimum and maximum allowable flux. These bounds are set according to the maximal value of the RMF(s) found in step (1), in general by setting the lower bound corresponding to each RMF to some fraction of its maximal value. The great majority of the lower and upper bounds do not correspond to an RMF and are set to the same value as in a standard FBA problem, usually to arbitrarily high and low values, but sometimes to finite values as for input constraints (glucose uptake, for example) and irreversible reactions.

The above optimization problem would generally be difficult to solve due to the presence of an absolute value operator, but in this case, a trivial simplification converts the above problem to a standard LP problem. Each reaction defined as possibly reversible (containing a negative lower bound) is converted to two irreversible reactions, thus restricting all fluxes to be positive, and removing the need for the absolute value.

In general, some reactions will not have available data. The algorithm takes a conservative approach and designates these reactions as active; hence the term “gene inactivation” is part of the method name. The algorithm treats these reactions as if they had data that surpassed the cutoff; this is a conservative approach to avoid any penalty for absent data. The lack of data does have implications for the interpretation of results. It is entirely possible that given better data, these reactions would be determined to be absent, perhaps necessitating the activation of other reactions. Clearly, with limited data, the results must be considered with caution. In general, this is far more of a concern for human metabolic networks than for *E. coli*.

### Context-Specific Networks for *E. coli*

We have used the GIMME algorithm to produce context-specific metabolic networks for *E. coli* for several different conditions and to compare inconsistency scores for different strains of the bacterium. We show that the inconsistency scores agree with experimental data in nearly all cases. Gene expression data from different conditions of *E. coli* growth are the input data, and the independent validation data is phenotypic data describing the relative growth and product secretion.

#### Strains adapted to novel substrates

Adaptive evolution has been used in the laboratory to improve the growth rate of *E. coli* on culture media to which it is unaccustomed [16]. Metabolic models of *E. coli* predict better growth than it initially achieves in the laboratory when it is given a substrate other than glucose to use as a carbon source for growth. However, after selective pressure is applied to maximize growth, the actual growth has been shown to improve to match the prediction, typically after a month or two of serial passage of cells [16]. Using the GIMME algorithm, we constructed context-specific metabolic networks for three types of strains described in [17]: (1) wild-type strains, (2) strains evolved to growth on glycerol, and (3) strains evolved to growth on lactate.

**Figure 3:**
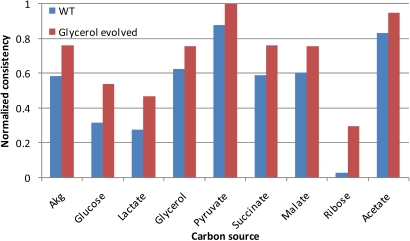
Glycerol-evolved strain normalized consistency scores. Normalized consistency scores are computed directly from the inconsistency scores, as described in the text. A higher normalized consistency score indicates that the gene expression data is relatively more consistent with the RMF. Thus, here the gene expression data from the glycerol-evolved strains are more consistent with highly efficient growth on each of the carbon sources tested. The p values, determined by permutation testing, are less than 0.01 in all cases here.

**Figure 4:**
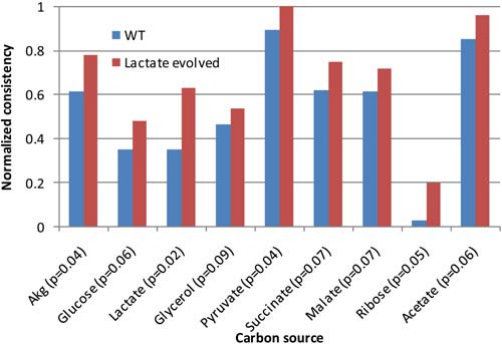
Lactate-evolved strain consistency scores. This figure demonstrates the same result as Figure 3, but with strains evolved on lactate. The normalized consistency scores for growth on each of the tested carbon sources are higher for evolved strains, indicating that the gene expression data from the evolved strains are more consistent with efficient growth on each carbon source.

The gene expression data used to construct the models consists of CEL files containing the data described in [17], normalized using GCRMA [14]. The data was mapped from genes to reactions using the gene-protein-reaction associations from the reconstruction [18]. The threshold (x_cutoff_) was set at 12, meaning that reactions assigned a normalized value greater than 12 are assumed to be present; similar results were noted at other thresholds. The RMF was growth on a given carbon source, and the context-specific metabolic networks were forced to grow no less than 90% of optimal growth. Because the evolved strains nearly always grow better than wild-type strains on a variety of carbon sources [19], metabolic networks for optimal growth on nine carbon sources were constructed. The results are shown in Figure 3 (glycerol evolved strains) and Figure 4 (lactate evolved strains). For these figures, the inconsistency scores were used to calculate normalized consistency scores (see Materials and Methods for details); a higher normalized consistency score indicates that the gene expression profile is more consistent with the objective. The figures show that the gene expression state of evolved strains are always more consistent with growth on the nine substrates, paralleling the phenotypic findings from [19] in nearly all cases. These findings demonstrate that the evolved strains have gene expression states that are more consistent (than wild type strains) with usage of the optimal networks for growth on a variety of carbon sources.

#### Metabolic engineering strain

**Figure 5:**
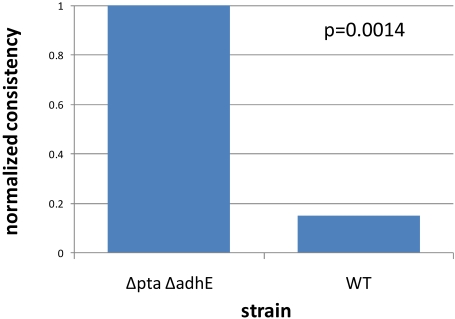
Metabolic engineering strain consistency score. The normalized consistency score for an *E. coli* strain designed to produce lactate indicate that the Δpta ΔadhE strain has a metabolic gene expression state consistent with the simultaneous production of lactate and growth when compared with the wild-type. This higher normalized consistency score indicates that the gene expression data from the double deletion strain is more consistent with the metabolic engineering objective than the wild-type strain, in accordance with experimental measurements.

Metabolic engineering seeks to optimize bacterial strains to produce a valuable product from a less expensive set of molecules. Rational design of strains for metabolic engineering is possible with genome-scale metabolic models [20]. Adaptive evolution of knock-out strains of *E. coli* can be used to optimize such strains [21]. We determined the inconsistency scores with GIMME for replicates of a Δpta ΔadhE strain that is designed to produce lactate as a byproduct of anaerobic growth on glucose, as described in [21], as well as wild-type strains. The objective used was growth and lactate production was fixed at a rate consistent with experimental data from [21]. As shown in Figure 5, the designed strain has gene expression data that is more consistent with growth-coupled lactate production, exactly as experimental data indicates. The gene deletions and subsequent evolution have led to a global metabolic gene expression state that is more consistent with growth-coupled lactate production than the wild-type strain.

We used this data set to verify the robustness of the algorithm to two different factors. First, we tested the effect of altering the cutoff by recalculating the results for cutoffs ranging from eight to 14, in increments of 0.1. We found that the consistency scores were significantly different for all cutoffs. For some cutoffs, the p value was not as good as for others, but p<0.01 for all choices of cutoff within the range tested. Second, we verified the robustness of the algorithm with a jackknife test. We randomly removed 5% of the expression values mapped to reactions 100 times and recomputed the context-specific networks and consistency scores. We found that for all repetitions the same conclusion was reached, although in some cases the p values were not quite as low as when using all of the data. In all cases, the conclusion was reached with p<0.02, which demonstrates slightly lower performance with all of the data available. This suggests that the algorithm should be expected to have greater statistical power when as many reactions as possible are assigned data.

#### Terminal electron acceptor effect on network

**Figure 6:**
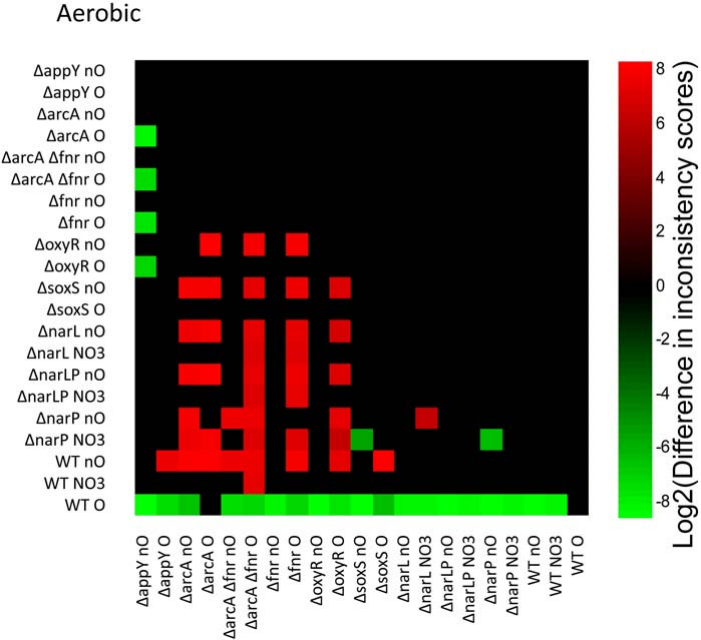
Pairwise comparisons of consistency for aerobic conditions. A graphical representation of the log2 transform of the difference between inconsistency scores. A green box indicates that the sample on the *y*-axis is more consistent with aerobic growth than the sample on the *x*-axis. Red boxes indicate the opposite. Differences that do not meet p<0.05 are left blank. The shade of red or green quantifies the log2 of the difference in inconsistency scores. The position of green and red blocks here indicates that in all statistically significant cases, strains grown with oxygen have gene expression more consistent with efficient aerobic growth than strains grown without oxygen.

**Figure 7:**
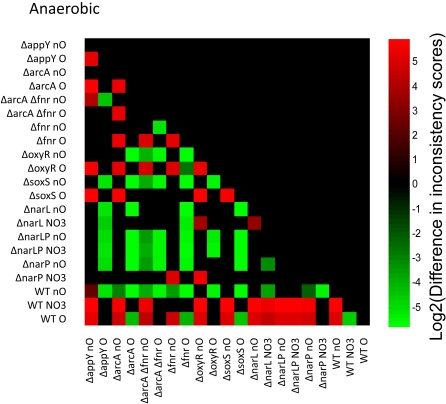
Pairwise comparisons of consistency for anaerobic conditions. A graphical representation of the log2 transform of the difference between inconsistency scores. A green box indicates that the sample on the *y*-axis is more consistent with anaerobic growth than the sample on the *x*-axis. Red boxes indicate the opposite. Differences that do not meet p<0.05 are left blank. The shade of red or green quantifies the log2 of the difference in inconsistency scores. The position of green and red blocks shows that in nearly all cases that are statistically significant, gene expression data for strains grown without oxygen is more consistent with efficient anaerobic growth than strains grown with oxygen.

**Figure 8:**
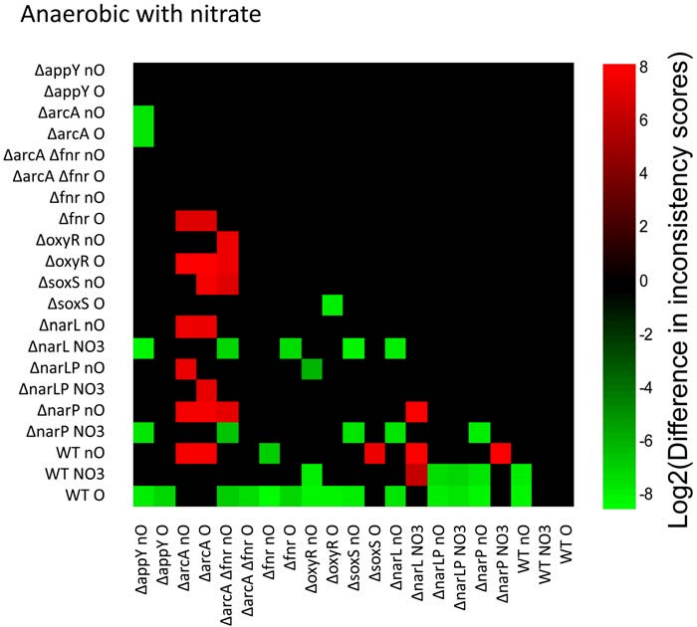
Pairwise comparisons of consistency for nitrate conditions. A graphical representation of the log2 transform of the difference between inconsistency scores. A green box indicates that the sample on the *y*-axis is more consistent with nitrate growth than the sample on the *x*-axis. Red boxes indicate the opposite. Differences that do not meet p<0.05 are left blank. The shade of red or green quantifies the log2 of the difference in inconsistency scores. The position of green and red blocks indicates that in most cases, gene expression from strains grown with nitrate as the terminal electron acceptor is more consistent with efficient growth under this condition than strains grown under other conditions.

The growth of *E. coli* varies depending on the availability of terminal electron acceptors, usually oxygen or nitrate. Gene expression data from a total of 21 different strain/electron acceptor conditions was analyzed to construct the most consistent models for growth with oxygen, without oxygen, and with nitrate. The expectation is that the strain data taken from a given condition (for example, aerobic) should be more consistent with growth on that condition (again, aerobic) than strain data from a different condition. Pairwise comparisons were made between all consistency scores, and the results are shown in Figures 6, 7, and 8, for aerobic, anaerobic, and anaerobic nitrate conditions, respectively. A green box indicates that the strain/condition indicated on the y axis is more consistent with growth than the strain on the x-axis; a red box indicates the opposite. The intensity of the green or red color indicates the difference in inconsistency scores, after log2 transformation for visualization scaling. Black boxes indicate that no statistically significant (p<0.05) conclusion could be reached from the data. In all cases where statistically significant conclusions were possible, gene expression in strains grown with oxygen is more consistent with aerobic growth than gene expression from strains grown without (Figure 6). In 99% of cases that are statistically significant, gene expression in strains grown anaerobically is more consistent with anaerobic growth than the data from strains grown with oxygen (Figure 7). The trend holds 90% of the time for anaerobic growth with nitrate (Figure 8). As would be expected, different subsets of reactions are active for each condition. Taken with the previous results, this provides strong support for the inconsistency scores that emerge from the algorithm and provides a positive control.

### Context-Specific Networks for Human Cells

The wide variety of human cell types in the body do not share a simple objective such as cellular growth, but rather have a multiplicity of functions necessary for multi-cellular life. Accordingly, understanding the metabolism of any particular cell type requires a model that contains only the reactions present in that cell type, without potentially thousands of extraneous reactions. Human Recon 1 [22] will contain many reactions that are inactive in particular cell types. Accurate models require their removal, and the GIMME algorithm provides a framework for this process. Herein we describe the first functional genome-scale metabolic models for particular human cells, in this case, skeletal muscle cells in different conditions.

#### Data sources

We used three publicly available sets of gene expression data for skeletal muscle cells, as depicted in Table 1.

**Table 1:** Datasets used to create context-specific skeletal muscle models.

Abbreviation	Description	Reference	GEO Accession Number
GB	3 patients before and 1 year after gastric bypass surgery (vastus lateralis).	[27]	GDS2089
GI	6 subjects before glucose/insulin infusion via clamp and 2 hours after beginning (vastus lateralis)	[28]	GSE7146
FO	24 subjects divided into 3 groups of eight: morbidly obese (MO), not obese (NO), and obese (O) (rectus abdominus).	[27]	GDS268

**Figure 9:**
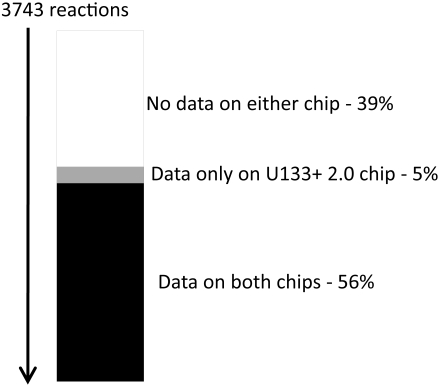
The mapping of Affymetrix gene chip data to reactions. Reactions in the white area have no usable gene chip data on either platform. Reactions in grey have usable data only on the 133+ 2.0 platform. Reactions in black have usable data for both the 133+ 2.0 and the 133A platform. Importantly, 5% (179) of the reactions are only represented on the 133+ 2.0 chip, potentially increasing scores across chips. The average difference score is 340, so a difference of 179 reactions is greater than a 50% impact.

These three datasets were originally gathered for purposes completely distinct from creating context-specific metabolic networks, just as the *E. coli* datasets described earlier were. Nevertheless, they can be interpreted in the context of a genome-scale metabolic network towards this end. All three datasets were collected using Affymetrix (Santa Clara, CA) gene expression arrays. The GB dataset used U133+ 2.0 arrays, while the GI and FO datasets used U133A arrays. While the arrays are similar, the U133+ 2.0 array is able to provide reliable trancriptomic data for 179 reactions beyond what the U133A array can provide. The coverage of these arrays in terms of model reactions is shown in Figure 9. Each probeset that corresponded to a metabolic gene was mapped to that gene, provided that the annotation information for that particular array type indicated that the probeset sequence was unique to either that gene or a closely related gene. Probesets with sequences that correspond to multiple, unrelated genes were ignored. The values associated with the expression of genes were mapped to reactions through the gene-protein-reaction associations, as described earlier and in Materials and Methods.

#### Model creation and comparison

**Figure 10:**
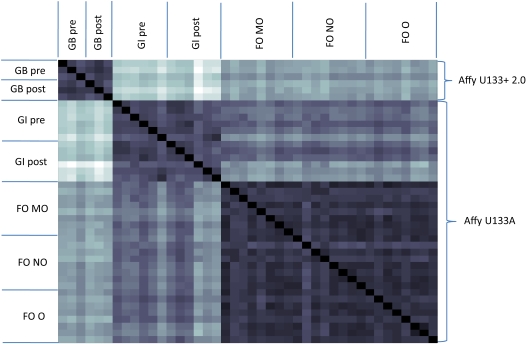
A comparison of skeletal muscle models. This heat map displays the level of difference in each pair of models. Darker squares represent models that are more similar to each other than lighter squares. A black square (as on the diagonal) indicates identical models, and a white square indicates the most different pair of models. The three darker blocks that surround the main diagonal are the comparisons of samples within each dataset to each other. These darker blocks show that the models within each dataset tend to be more similar to each other than to models from other datasets. The models from a particular expression array type also appear to be more similar to each other than to models from a different array types, but the data available do not allow us to show that this is actually true, as is shown in Figure 11.

**Figure 11:**
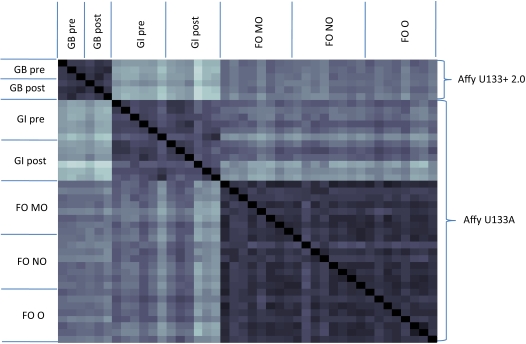
A comparison of skeletal muscle models, using only reactions that have data for both gene chips used. This figure is the same as Figure 10, but the distances that are graphically represented are computed using only reactions that have data on both types of gene chips. The 5% of reactions that are represented on the U133+ 2.0 chip but not the U133A chip are not used for comparison. Here we no longer see any bias based on chip type, but rather we see that the FO datasets appear to be similar to both GI and GB data sets, instead of just the GI data sets. The chip type does not appear to affect the distances as much as the different experiments do. The chip type does appear to affect the reactions that the algorithm defines as active versus inactive, as seen in the differences between Figures 10 and 11.

Each of the 42 (6+12+24) gene expression datasets was used with the GIMME algorithm to create a model that produces ATP at no less than half the optimal efficiency and matches the data as closely as possible. These models were compared on a pairwise basis by finding the number of reactions that are different in the two models under comparison. On average, two models differ by 340 reactions, which is approximately 10% of the reactions in the global model. The pairwise distances are shown graphically in Figure 10. Darker squares represent pairs of networks that are more similar than lighter squares. Two trends are immediately apparent. First, the metabolic networks that are derived from each dataset are more similar to others derived from that same dataset, as shown by the three large dark squares that surround the diagonal. Secondly, it appears that the GI and FO models are more similar to each other than to the GB models. Initially, we suspected that the gene chip might bias this result, so we recomputed the distance between each pair of models, ignoring the 179 reactions that are not present on the U133A array. This result is graphically depicted in Figure 11, showing that the FO models are similar to both the GB and GI models, but the GI models are not similar to the GB models. Comparing models generated with different gene expression platforms must be done with caution. The bottom line is that there are 179 metabolic reactions that the GB models could elect not to use based on data, but the GI and FO models cannot because no data is present; the GIMME procedure will only turn off a reaction in the presence of some data mapped to that reaction. Better coverage of metabolic reactions on gene expression arrays will lead to fewer extraneous reactions in resulting models.

#### Two significant results

In spite of the difficulties of comparing models derived from different sources, two statistically significant differences emerge from the analysis. First, a given patient is more similar to himself before and after either gastric bypass or glucose/insulin infusion than he is to other patients. We took the similarity scores for the GB and GI patients and created two separate groups: (A) all matched patients before and after and (B) all unmatched patients from the same dataset. Permutation testing demonstrated that group A has a smaller mean distance than group B (p<0.01). Secondly, we looked at consistency scores, asking if any group was more consistent with high ATP production than any other. Only one statistically significant (p<0.01) result emerged, that the after-GI patients are more consistent with high ATP production than the before-GI patients. Again, this result is exactly as expected; muscle cells that have been given a substantial dose of glucose and insulin in the bloodstream should be more consistent with high ATP production.

### Conclusions

The work reported herein details the first available method to both produce a guaranteed functional metabolic model specific to a set of gene expression data and quantify the agreement between gene expression data and one or more metabolic objectives. We have demonstrated the functionality of this GIMME method with gene expression data from *E. coli* and human skeletal muscle cells. We have shown that (1) the computed consistency between gene expression data for different conditions and RMF agrees with physiological data, (2) the most consistent networks depend on the metabolic objective and media conditions, and (3) the most consistent networks for human skeletal muscle cells contain significantly fewer reactions than the global human model.

Initially, we expected that the results for human models would be more interesting than those for any other organism reconstructed to date, principally because we expected that human cells would show the most variability across conditions. However, the lack of available data for a substantial number of human metabolic reactions confuses attempts at comparison. We showed that reducing the number of reactions considered by 5% can change the apparent differences between different datasets. In addition, the lack of replicates in human gene expression data sets and the difficulty in obtaining high quality biological controls complicates matters and reduces the statistical power of comparisons. We have higher confidence in the results presented for *E. coli* because nearly all of the gene-associated reactions have data available, replicates are available, and controls are present. We also found that a substantial number of reactions in *E. coli* do vary in activity when different input conditions are provided. In the end, we conclude that a tool originally conceived to plug a key gap in the analysis of human cellular metabolism actually provides more immediate use in the analysis of microbial metabolism.

With metabolic reconstructions growing in size and becoming available for more and more organisms, tools to filter global reaction lists into context-specific reaction lists will be highly useful. Meaningful analysis of the human metabolic network will require procedures such as GIMME in order to accurately predict phenotypes.

## Materials and Methods

The metabolic networks for *E. coli* and human cells were imported into Matlab with the COBRA Toolbox [23]. The gene expression data for *E. coli* has been obtained by our lab and is previously published as cited above. The gene expression data for human skeletal muscle cells was downloaded from NCBI GEO [24] as CEL files.

### Gene Expression Data Processing

The gene expression data was obtained as CEL files and processed using Bioconductor [25]. The data for *E. coli* was processed using GCRMA as implemented within Bioconductor [14]. The data for human skeletal muscle was processed using the affy package [26] and the mas5calls function. The p values were subtracted from 1 and the resulting value used as a quantitative measure of likelihood that the gene was available. The default parameters were used. For all datasets, the expression level of each reaction was determined by mapping any available data from genes associated with that reaction. If data was not available for any gene associated with a reaction, it was given a score of −1. If data was available for one or more genes, a single score was computed by evaluating the boolean GPR associations; OR's would evaluate to the greater of the two values, AND's to the lesser. The end result was a score for each reaction from each set of data, either −1 or non-negative, with greater numbers implying greater certainty that reaction is present. This is the data that was input into the GIMME algorithm to compute the consistency scores and context-specific metabolic networks.

### GIMME Implementation

The GIMME algorithm is implemented in Matlab, using functions in the COBRA Toolbox. In general, any robust linear programming solver should work; we used Tomlab (Tomlab Optimization, Pullman, WA).

### Conversion to Normalized Consistency Scores

The output from the GIMME algorithm is an inconsistency score, and a higher score means that the gene expression data is less consistent with the model achieving the desired objective. For visualization purposes only, these scores are converted into normalized consistency scores, with a higher score indicating greater consistency between the data and the modal achieving the objective. For a given set of scores, each inconsistency score is subtracted from 1.02 * (maximum inconsistency score) to produce a set of consistency scores. Each consistency score is divided by the maximum consistency score to produce a set of normalized consistency scores. The 1.02 factor assures that the smallest consistency score is slightly greater than zero and easy to visualize on a graph.

### Statistical Significance Testing

Permutation testing with 10,000 randomizations was used to determine the statistical significance of all results with regard to consistency scores. This testing was implemented in Matlab.

### Visualization

Heat-map type representations were produced in Matlab. Other graphs were produced in Excel (Microsoft, Redmond, WA).
